# Cross-sectional survey evaluating blood pressure control ACHIEVEment in hypertensive patients treated with multiple anti-hypertensive agents in Belgium and Luxembourg

**DOI:** 10.1371/journal.pone.0206510

**Published:** 2018-11-01

**Authors:** Marc Leeman, Michèle Dramaix, Bregt Van Nieuwenhuyse, Josse R. Thomas

**Affiliations:** 1 Department of Internal Medicine and Hypertension Clinic, Erasme University Hospital, Université libre de Bruxelles, Brussels, Belgium; 2 Research Centre of Epidemiology, Biostatistics and Clinical Research, School of Public Health, Université libre de Bruxelles, Brussels, Belgium; 3 Servier Benelux Scientific Department, Brussels, Belgium; 4 PharmaCS, Merchtem, Belgium; International University of Health and Welfare, School of Medicine, JAPAN

## Abstract

**Objective:**

This study evaluates the actual blood pressure control rate and its estimation by general practitioners, the use of single-pill or free combinations, and the attitude towards single-pill combinations in primary care.

**Methods:**

Cross-sectional observational survey in primary care between January 2015 and September 2016 in Belgium and Luxembourg. The participating general practitioners enrolled hypertensive patients taking at least 2 antihypertensive molecules (as fixed or free associations).

**Results:**

742 general practitioners included a total of 8,006 patients, with a mean age of 66 ± 12 years. Systolic blood pressure and diastolic blood pressure were respectively 141 ± 17 mmHg and 82 ± 10 mmHg (means ± SD). These patients had a blood pressure control rate of 45%, whereas it was estimated by general practitioners to be 60%. General practitioners with 11–25 years’ experience performed better than general practitioners with 36–51 years’ experience in the evaluation of blood pressure control. Combinations used were free in 39%, single-pill in 34% and mixed in 27% of the patients. Patients receiving single-pill combinations were younger than those treated with free combinations (63 ± 12 vs. 68 ± 12 years, p < 0.001), with fewer comorbidities (39 vs. 55%, p < 0.001). In patients treated solely with free pill associations, 66% of patient cases, general practitioners were willing to switch to a single-pill combination. The main reasons were improved adherence (76%) and better blood pressure control (64%).

**Conclusion:**

In patients requiring at least two antihypertensive drugs, blood pressure control rate remains low and is overestimated by general practitioners. Free combinations remain largely used although many general practitioners seem willing to shift to single-pill combinations. Treatment simplification could improve adherence and blood pressure control rate, which has been shown to lead to reduced morbidity and mortality.

## Introduction

Arterial hypertension is one of the main modifiable risk factors for cardiovascular diseases worldwide [1, 2]. On top of non-pharmacological measures to prevent and to treat arterial hypertension, the 2013 guidelines of the European Society of Hypertension and the European Society of Cardiology (ESH-ESC) recommend the use of antihypertensive drugs [1]. The different blood pressure-lowering drug classes currently available, e.g. diuretics, beta-blockers, calcium antagonists, angiotensin-converting enzyme inhibitors and angiotensin receptor blockers, are all suitable for the treatment of high blood pressure, either as monotherapy or in selected combinations [1]. Since monotherapy is only effective in a limited number of patients, the use of combination therapy is recommended because it is more effective and better tolerated than high-dose monotherapy. Combination therapy could even be considered as the initial approach in patients with marked blood pressure elevation and/or high cardiovascular risk [1].

Despite available treatments, control of arterial hypertension remains suboptimal [3], which is largely due to suboptimal treatment. It has been shown that more than 50% of the treated patients receive an inappropriate prescription [4]. Inappropriate use of antihypertensive agents is particularly important when patients are treated with more than one antihypertensive agent. Up to 40% of patients treated with combination therapy receive inappropriate combinations or dosages [5].

When combination therapy is used, the use of single-pill drug combinations (SPCs), also known as fixed-dose combinations, is favoured by guidelines [1], because reducing the number of tablets to be taken daily improves adherence and blood pressure control [1, 6]. In the light of these European recommendations and the wide availability and current experience with combinations treatment in hypertension, several scientific societies have updated their recommendations to include SPCs as the initial treatment in newly diagnosed hypertensive patients [7–9]. Initial combination therapy with antihypertensive agents from different classes from the start of hypertension treatment may carry several important advantages such as faster blood pressure control, better cardiovascular protection, long-term achievement of target blood pressure values and better adherence [10].

In Belgium and Luxembourg, epidemiological data on the use of antihypertensive drug combinations in primary care are scarce. The current study was conducted in these 2 countries in a sample of patients to whom at least 2 antihypertensive agents were prescribed. The objectives of this study were to evaluate actual blood pressure control, its estimation by general practitioners (GPs), the type of drug combinations used and the GPs’ attitude towards the prescription of SPCs.

## Materials and methods

### Study design

The complete study protocol can be found on dx.doi.org/10.17504/protocols.io.tkwekxe

For this observational cross-sectional survey, a random sample of 840 primary care physicians throughout Belgium and Luxembourg were contacted to participate. From January 2015 to September 2016, each physician included 12 consecutive hypertensive patients treated with at least 2 antihypertensive drugs, in order to obtain a planned study sample of 10,000 patients. In total 742 GPs (88% of the intended sample) included 8,006 (80% of the intended sample) patients treated with at least 2 antihypertensive drugs, resulting in a capture rate of on average 10.8 included patients per participating GP.

Paper case report forms were filled out manually with existing patient data recently registered in the medical file of the participating GPs as part of their routine clinical practice. The following data were collected for each patient: demographics (age and sex), current office systolic and diastolic blood pressure, doctors’ judgement about blood pressure control, presence of comorbidities (diabetes mellitus, history of a previous cardiovascular event, renal insufficiency or other), type of prescribed antihypertensive drugs (SPC, free combination, or mixed SPC and free combination), and GPs willingness to switch to SPCs.

No specific recommendations were given regarding the BP measurements and the definitions of co-morbidities were left to the discretion of the GPs.

### Definitions

Systolic blood pressure and diastolic blood pressure cut-off values were based on the 2013 ESH-ESC guidelines [1]. For the whole analysis, blood pressure control was defined as a systolic blood pressure < 140 mmHg and a diastolic blood pressure < 90 mmHg, or < 85 mmHg in patients with diabetes mellitus. Estimation of the blood pressure control by the GP was based on the GPs routine clinical diagnosis rules and determined only with the question “is your patient controlled?” which could be answered with YES/NO. A separate analysis was performed for the population of patients aged ≥ 80 years. For these patients, the 2013 ESH-ESC guidelines advocate for a systolic blood pressure between 140 and 150 mmHg. Hence, in the analysis of the octogenarians, systolic blood pressure control was defined as a systolic blood pressure < 150 mmHg.

### Ethical aspects

Given the retrospective observational design of the study, no ethics committee approval was requested, in accordance with the Belgian law on experiments involving human subjects (7 May 2004). The study was conducted according to the quality standards for non-interventional studies outlined in the prevailing Code of Deontology of the Belgian pharmaceutical industry association (pharma.be).

### Statistical analysis

All data collected in the case report forms were entered in an Excel table. In order to verify the quality of the data entry, a quality control was performed on the concordance of the data of 5% of the patients, randomly selected by a computer algorithm, between the Excel table and the original case report forms. In this 5% subsample of the dataset, 0% errors in data entry were identified. The statistical analysis was subsequently performed in this controlled dataset using Stata software v. 14. All statistical tests were two-sided using a significance level of 0.001. Data is are presented as mean ± standard deviation or as proportions (%). Student’s t-test and one-way ANOVA were applied to compare means and Chi-squared test was used to compare proportions.

## Results

### Study sample and demographics (Table 1)

Of the 840 GPs contacted for this study, a total of 742 GPs enrolled 8,006 hypertensive patients treated with at least 2 antihypertensive drugs. The mean age was 66 ± 12 years and 54% of the patients were male. Mean systolic and diastolic blood pressure were 141 ± 17 mm Hg and 82 ± 10 mm Hg, respectively. No difference in mean blood pressure were observed in the different subgroups of patients. Comorbidities were observed in 64% of the patients. Among the patients with comorbidities, 49% had diabetes mellitus, 50% had a previous cardiovascular event, 16% had renal insufficiency and 19% had other comorbidities. Patients were treated with either two (57%), three (31%) or more than three (12%) antihypertensive drugs. Combinations were free in 39%, single-pill in 34% and mixed in 27% of the patients. There was no significant difference observed in use of SPCs between Belgium and Luxembourg.

**Table 1:** Population demographics.

DEMOGRAPHICS	
n	8006
Age (years, ± SD)	66 ± 12
Men (%)	54
SBP (mm Hg, ± SD)	141 ± 17
DBP (mm Hg, ± SD)	82 ± 10
COMORBIDITIES	
Any type of comorbidity (%)	64 (n = 5155)
Diabetes mellitus (%)*	49 (n = 2545)
Prior CV event (%)*	50 (n = 2563)
Renal insufficiency (%)*	16 (n = 826)
Other (%)*	18 (n = 964)
NUMBER OF ANTI-HYPERTENSIVE AGENTS	
2 anti-HT agents (%)	57
3 anti-HT agents (%)	31
> 3 anti-HT agents	12
TREATMENT REGIMEN	
Free associations (%)	39
Free associations combined with SPCs (%)	27
SPCs (%)	34

SBP, systolic blood pressure; DBP, diastolic blood pressure; CV, cardiovascular; anti-HT, anti-hypertensive; SPCs, single-pill combinations.

*percentages of types of comorbidities is % out of population presenting any type of comorbidity

### Type of combinations

SPCs were exclusively used in only 34% of patients, while in the remaining 66% of patients the combinations used were either free (39%) or comprised both SPC and free components (27%). Patients taking SPCs were younger, were more often men and had a lower prevalence of comorbidities (Table 2).

**Table 2:** Population demographics according to prescribed treatment regimen.

	SPCs	Free associations	Free associations + SPC	P
% of study population	34	39	27	
Age (years, ± SD)	63 ± 12	68 ± 12	68 ± 12	< 0.001
Men (%)	56	54	52	< 0.001
COMORBIDITIES				
Any type of comorbidity (%)	54	71	68	< 0.001
Diabetes mellitus (%)*	54	46	50	< 0.001
Prior CV event (%)*	39	55	53	< 0.001
Renal insufficiency (%)*	11	19	16	< 0.001
Other (%)	21	17	19	NS

SPCs, single-pill combinations. P-value refers to between group differences between either “SPC”, “Free associations” or “Free associations + SPCs”; NS: not significant.

*percentages of types of comorbidities is % out of population presenting any type of comorbidity

### Actual versus estimated blood pressure control rate

**Fig 1:**
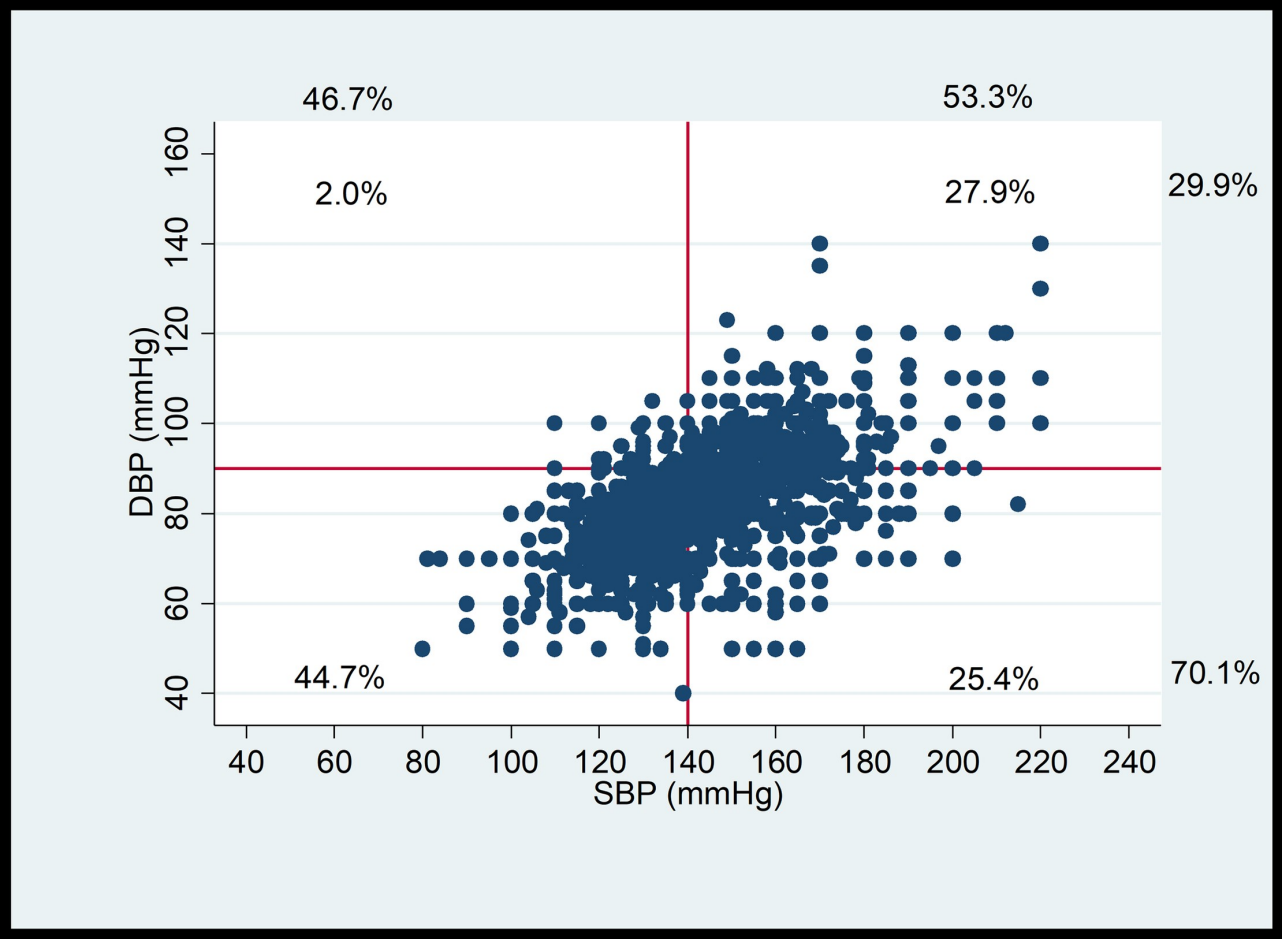
Blood pressure distribution in the entire study population. Systolic and diastolic blood pressure of the 8006 hypertensive patients treated with at least 2 antihypertensive drugs. Lines represent the upper limit of target blood pressure. Percentages in margin indicate the proportion of patients above and below the limit. Percentages within the graph indicate the proportion of individuals in a given quadrant. SBP: systolic blood pressure; DBP: diastolic blood pressure.

Control of both systolic and diastolic blood pressure based on the 2013 ESH-ESC guidelines was observed in 45% of the patients. Systolic blood pressure was controlled in 47% and diastolic blood pressure in 70% of the patients (Fig 1).

**Fig 2:**
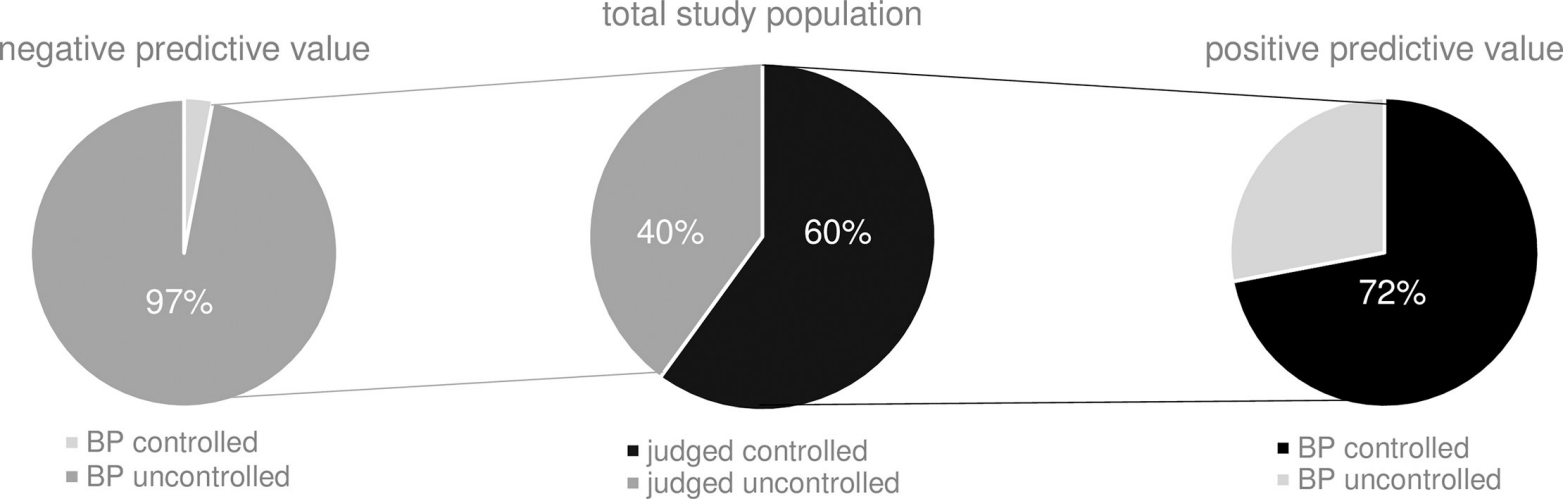
GPs performance in blood pressure control judgement in patients treated with at least 2 antihypertensive drugs. Positive and negative predictive value GP judgement. BP: blood pressure.

The blood pressure control rate was estimated by the GPs to be 60%. When a patient was considered by the GPs as having controlled blood pressure, this patient was truly controlled in 72% of the cases (positive predictive value). On the other hand, when a patient was estimated to have his blood pressure uncontrolled, this statement was correct in 97% of the cases (negative predictive value) (Fig 2).

**Fig 3:**
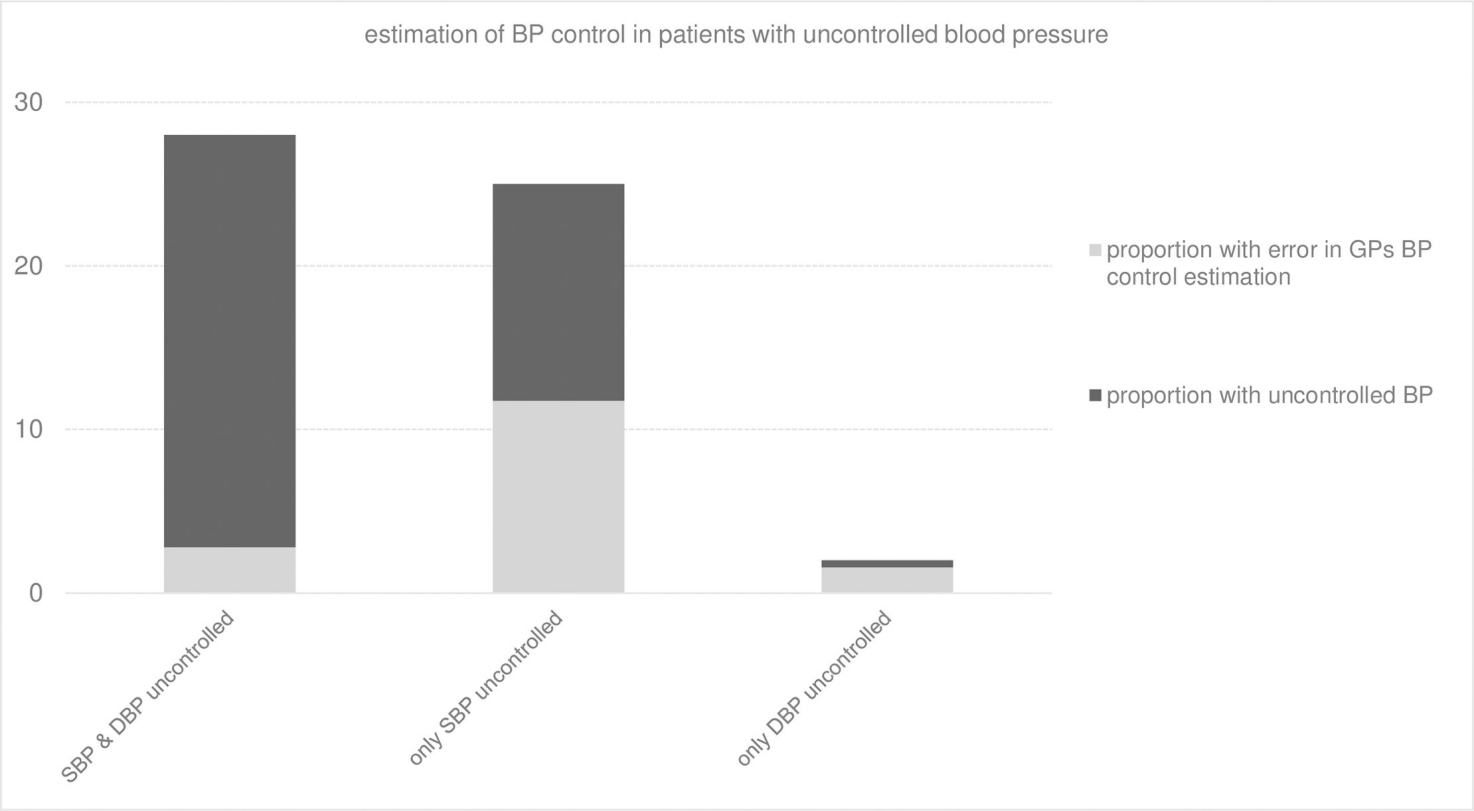
Estimation of blood pressure control in patients with uncontrolled blood pressure. BP: blood pressure; SBP: systolic blood pressure; DBP: diastolic blood pressure; GP: general practitioner.

When both systolic and diastolic blood pressure were uncontrolled (28% of the study population), GPs wrongly considered the blood pressure to be controlled in 10% of the cases. When only systolic blood pressure was not controlled (25% of the study population), 47% of the patients were erroneously estimated to be controlled. When only diastolic blood pressure was not controlled (2% of the study population), 78% of the patients were wrongly estimated to be controlled (Fig 3).

Best concordance between the measured blood pressure control rate based on Guidelines and the blood pressure control rate evaluated by the GP was observed in GPs with 11–25 years’ experience (kappa coefficient 0.70, 95% confidence interval 0.67–0.73) and worst in older GPs with 36–51 years’ experience (kappa coefficient 0.54, 95% confidence interval 0.47–0.57).

### Willingness to switch to SPCs

In the population of patients solely treated with free pill associations (n = 3,087), doctors were willing to switch to a SPC in 66% of the cases. Of these cases when doctors were willing to switch to a SPC, in 55% of the cases the preferred switch was to a SPC concerned two-drug combination, in 37% of the cases a SPC concerned three-drug combinations and in 8% either single pill, two-drug or three-drug therapies.

**Fig 4:**
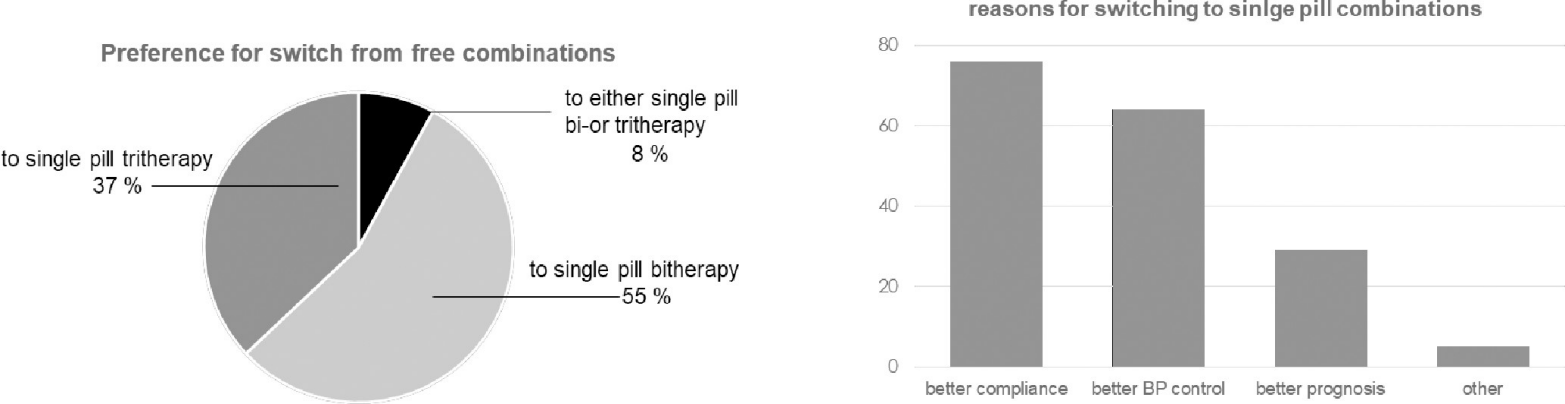
Willingness of GPs to switch towards single-pill combinations and main reasons.

The main reasons for which GPs considered shifting to SPCs were improved adherence (76%) and better blood pressure control (64%) (Fig 4).

### Data on octogenarians (Table 3)

In our study population, 1,263 (16%) of the patients were aged 80 years or more, with more women (58%) than men. Octogenarians had more comorbidities than patients aged less than 80 years. The most prevalent comorbidities in octogenarians in comparison with patients under 80 years were renal insufficiency and prior cardiovascular event, whereas diabetes and other comorbidities were less frequent. Octogenarians were more often treated with free drug combinations than younger patients (48% vs. 37%, p< 0.001).

**Table 3:** Demographics of octogenarians versus non-octogenarians.

	≥ 80 years	< 80 years	P
% of study population	16	84	
Age (years, ± SD)	84 ± 10	63 ± 10	< 0.001
Men (%)	42	57	< 0.001
COMORBIDITIES			
Any type of comorbidity (%)	76	62	< 0.001
Diabetes mellitus (%)*	38	52	< 0.001
Prior CV event (%)*	63	46	< 0.001
Renal insufficiency (%)*	31	11	< 0.001
Other (%)*	15	20	< 0.001
TREATMENT REGIMEN			
Free associations (%)	48	37	< 0.001
Free associations combined with SPCs (%)	31	27	< 0.001
SPCs (%)	21	37	< 0.001

SPCs, single-pill combinations. P-value refers to between group differences between ≥ 80 years and < 80 years; NS: not significant.

*percentages of types of comorbidities is % out of population presenting any type of comorbidity

Based on the 2013 ESH-ESC criteria (systolic blood pressure < 150 mmHg and diastolic blood pressure < 90 mmHg), the actual blood pressure control rate in octogenarians was 71%. When blood pressure control rate was defined as a blood pressure < 140/90 mmHg, it was 50% in octogenarians compared to 44% in patients aged less than 80 years (P < 0.001). In octogenarians, GPs considered blood pressure as controlled in 68% of the patients. When an octogenarian was judged by the GP as having controlled blood pressure, this patient was truly controlled in only 72% of the cases, and when an octogenarian was judged as having uncontrolled blood pressure, this statement was correct in 96% of the cases (no significant difference compared to patients aged < 80 years).

## Discussion

In this large and specific population of hypertensive patients taking at least 2 antihypertensive drugs and followed in primary care, we observed a blood pressure control rate of 45%, which was overestimated by the GPs. Although the exclusive use of SPCs is relatively low compared to the use of combination treatment with at least one free component, a majority of GPs considered switching the therapy to SPCs in order to increase therapeutic adherence and blood pressure control.

Few studies have examined the blood pressure control rate in hypertensive patients in Belgium. Comparing two large epidemiological studies, blood pressure control rates of 25% and 33% were reported in treated hypertensive patients [11]. In 3,761 hypertensive patients followed in primary care, blood pressure control rate was 38% in treated patients [12]. In a large series of 11,562 treated patients, blood pressure control rate was 22% [13]. More recently, in 10,078 hypertensive patients from Belgium and Luxembourg, it was reported that 44% of the treated patients had blood pressure < 140/90 mmHg [14], a value close to the one of this study, performed in patients taking at least 2 antihypertensive drugs.

The large Prospective Urban Epidemiology (PURE) study examined hypertension prevalence, awareness, treatment and control in adult subjects from countries with different economic levels. Data were collected from January 2003 to December 2009. Among the patients treated for high blood pressure, 32.5% (7,634 patients) had their blood pressure controlled (95% confidence interval 31.9–33.1%). Control rate was 40.7% in high-income countries, and, specifically in Europe and North America, it was 38.5% [15]. In an extensive literature search including 135 population-based studies of almost one million adults from 90 countries worldwide, blood pressure control rate in high-income countries increased from 38.6% to 50.4% from 2000 to 2010 among subjects self-reporting antihypertensive treatment [16]. The EURIKA cross-sectional study included 7,641 adult outpatients with at least one major cardiovascular risk factor, selected from 12 European countries in 2009. Data were collected from May 2009 to January 2010. In treated hypertensive patients, overall control rate was 38.8%, and, specifically in Belgium, control rate was 43.7% [17]. The control rate observed in the present survey is in accordance with those reported in other studies, and could reflect improvement compared to earlier studies [11, 13, 16, 17].

As previously described, diastolic blood pressure is more often controlled than systolic blood pressure [13, 14]. Overall, despite the improvement compared to 2008, our study adds to the evidence that blood pressure control remains suboptimal in hypertensive patients. In the present study, we compared the actual blood pressure control rate with the blood pressure control rate as estimated by GPs. We found that blood pressure control rate was overestimated by GPs. More specifically, when GPs considered the blood pressure as controlled, this was true in only 72% of the patients. A potential implication of this finding is that GPs may not intensify antihypertensive therapy in many patients who would require it, and who therefore remain undertreated. This phenomenon, called therapeutic inertia, is a cause of poor blood pressure control rate. The European SHARE survey showed that physicians were “concerned” with a blood pressure of 149/92 mmHg and would “take immediate action” (i.e. intensify therapy) with a blood pressure of 168/100 mm Hg [18]. In a recent large study conducted in Belgium and Luxembourg, treatment intensification was planned in only 29% of the patients with systolic blood pressure remaining above 140 mmHg [14]. Interestingly, in our study practitioners with 11–25 years’ experience performed better than older ones with 36–51 years’ experience in the evaluation of blood pressure control rate, which emphasizes the need for continuous medical education.

SPCs were exclusively used in only 34% of patients, while in the remaining 66% of patients the combinations used were either free (39%) or comprised both SPC and free components (27%). The use of SPCs, as compared to their free-drug counterparts, has been shown to be associated with a significant higher adherence rate to treatment [19–22] and lower health-care costs [20], while a non-significant reduction in blood pressure [19] but a significant improved blood pressure control rate [21, 23] were reported. High adherence to antihypertensive treatment has been shown to be related to lower cardiovascular events [24–27]. A recent large population-based retrospective cohort study of 13,350 elderly patients (> 66 years) conducted in Ontario compared the effect of newly initiated antihypertensive treatment with a SPC versus a multi-pill therapy on a composite primary outcome of death or hospitalization for myocardial infarction, heart failure or stroke [27]. Patients initiated with a SPC had a significantly higher proportion of total follow-up days covered with medications and a lower frequency of the primary outcome [27]. Recently also, it has been shown, in a large group of Australian hypertensive patients, that treatment with SPCs resulted in a lower mortality compared to patients taking the same antihypertensive agents in separate pills [28]. We found that, in accordance with the 2013 ESH-ESC guidelines [1], many GPs consider switching from free combinations to SPCs, with the hope of improving therapeutic compliance and blood pressure control. A similar attitude was observed in another study where GPs planned to simplify antihypertensive therapy in 25% of the patients, with no change in doses [14].

Although the methodology of this study was not designed to identify the motivations of drug prescriptions by the GPs, we can speculate on several reasons. First, fixed dosages of the components into the SPC could limit adaptation of the dosages of the components, although, at least in Belgium and in Luxembourg, most SPC are launched with all dosages of the individual components. Second, until recently, European guidelines have recommended an antihypertensive strategy based on an initial monotherapy, followed, if BP target is not achieved, by the addition of other drugs [1]. Once the treatment adjusted, some GPs could not switch to SPCs for several reasons, including patient choice, fear of the consequence of poor compliance (one SPC missed means 2 or 3 drugs missed) or insufficient knowledge about the advantages of SPC [10]. Third, GPs could believe that the cost of SPCs is higher than the one of free combinations. Of note, in the Belgian market, a hypertension treatment with SPCs comes at a comparable or lower price compared to free combinations. For example the daily price for a treatment with a branded SPC of perindopril, indapamide and amlodipine at its highest dosage costs 0.71€/day, whereas the cheapest possible free combination of this three-drug therapy costs 0.77€/day. This also holds for low dosages and two-drug therapies, where for example a branded SPC of perindopril and amlodipine at its lowest dosage costs 0.33€/day, whereas the cheapest possible free combination of this two-drug therapy costs 0.36€/day.

Several initiatives have been proposed to improve BP control rate, including a more generalized prescription of SPC [29]. Based on an extensive review of the literature, the last ESH/ESC now clearly recommend to initiate treatment with a two-drug combination, preferably in a SPC (a grade I recommendation) [7]. Initiating treatment with a SPC is associated with an improved adherence and a faster blood pressure reduction, with possible favorable psychological impact on the patient, which may translate into a greater reduction in cardiovascular events [24–27]. Following the publication of this landmark document [7], we believe that national hypertension societies and health care organisms have the task to largely disseminate these recommendations among GPs, eventually after adaptation at the national level [29].

The inclusion of 1,263 patients aged ≥ 80 years allows us to describe, for the first time to our knowledge, the use of antihypertensive drug combinations in octogenarians. Expectedly, octogenarians were more often women and had more comorbidities than patients aged < 80 years. Octogenarians received more often free drug combinations. It is possible that GPs want to keep dosage adjustment flexibility in these more vulnerable patients with a high prevalence of kidney disease. On the other hand, SPC were more often used in younger patients with less comorbidities, presumably because GPs could believe that these patients require less “fine-tuning” of the dosages of the components in the SPC. Results from a recently published study from Germany are in accordance with this latter observation [22]. An analysis was performed in 81,958 patients receiving a combination of amlodipine and of an inhibitor of the renin-angiotensin-aldosterone system, either in a SPC or in free doses. Patients receiving the SPC were significantly younger and had less comorbidities [22].

This study has several limitations. Physicians participated to the study on a voluntary basis, which may reflect a particular interest in the management of high blood pressure. Therefore, they could be unrepresentative of the general GP population and their patients may not fully reflect the general population of hypertensive patients in Belgium and Luxembourg. No specific definitions were given for diabetes mellitus, previous cardiovascular events, renal insufficiency and other comorbidities, and given the retrospective nature of the study no specific recommendations were given for blood pressure measurement. Thresholds for blood pressure control were not recalled: overestimation of blood pressure control by GPs could reflect their lack of knowledge of the most recent guidelines. Results are based on treatment prescribed by the GPs, and it is inherent to the observational study design that we could not check whether the patients actually take the antihypertensive therapy which might have an influence as adherence to antihypertensive therapy is known to be low in hypertensive patients [6]. Finally, measurement of blood pressure, evaluation of blood pressure control by GPs and their plan for future prescriptions were based on data from one single consultation, which may not be representative of the usual condition of the patients and attitude of the GPs.

## Conclusion

In conclusion, in this large-scale study conducted in hypertensive patients treated with drug combinations, we observed a blood pressure control rate of 45%. The blood pressure control rate was overestimated by GPs, which could prevent treatment intensification in many patients (i.e. therapeutic inertia). Although the use of SPCs, free and mixed combinations was balanced in the study population, many GPs planned to substitute free combinations for SPC, which could lead to improved adherence, blood pressure control rate and hopefully better cardiovascular prognosis.

## Supporting information

S1 File Protocole scientifique ACHIEVE.pdf. Original scientific protocol of the ACHIEVE study.
(PDF)

S2 File Amendement_Ex. Investigateur. Amendment to the ACHIEVE protocol justifying the 2^nd^ inclusion wave of patients.
(PDF)

S3 File SCIENTIFIC PROTOCOL ACHIEVE_ENG. Scientific protocol and CRF of the ACIEVE study in english.
(PDF)

S4 File TREND checklist ACHIEVE.pdf. TREND statement checklist.
(PDF)

S1 Dataset S1.minimal anonymized data set ACHIEVE.zip. The anonymized dataset of the ACHIEVE study.
(ZIP)
