# Supplementary material for: Cross-sectional survey evaluating blood pressure control ACHIEVEment in hypertensive patients treated with multiple anti-hypertensive agents in Belgium and Luxembourg
Source: PLoS One. 2018 Nov 1;13(11):e0206510. doi: 10.1371/journal.pone.0206510 (PMC6211697; doi:10.1371/journal.pone.0206510)
Supplement: S1 File — Original scientific protocol of the ACHIEVE study. (PDF) [file pone.0206510.s001.pdf]

ACHIEVE

Etude observationnelle transversale du taux de contrôle tensionnel dans la population générale de patients hypertendus en Belgique traités par plusieurs antihypertenseurs suivant le type de traitement reçu

Protocole d'étude

1. Rationnel scientifique

L'hypertension artérielle reste toujours une cause majeure de mortalité et un des principaux facteurs de risque influençables des maladies cardio-, cérébro- et rénovasculaires. Malgré l'impact de cette maladie sur la santé publique et les coûts de soins, son diagnostic est souvent ignoré et son traitement reste sous-optimal. Il est estimé qu'au moins la moitié des patients hypertendus est méconnue, qu'au moins la moitié des patients traités reste non-contrôlée, et que la moitié des patients traités par antihypertenseurs n'adhère pas à son traitement à long terme.
Les médecins-généralistes jouent un rôle primordial dans son diagnostic précoce et son traitement adéquat.

Outre les mesures non-pharmacologiques pour la prévention et le traitement de l'hypertension, les recommandations récentes de la Société Européenne d'Hypertension et de la Société Européenne de Cardiologie de 2013(1) préconisent de rapidement débuter un traitement pharmacologique.

La prise en charge pharmacologique utilise les classes d'antihypertenseurs actuellement disponibles, c-à-d les diurétiques thiazidiques, les bêtabloquants, les antagonistes calciques, les inhibiteurs de l'enzyme de conversion et les antagonistes des récepteurs de l'angiotensine. En plus, ces recommandations mettent en avant l'importance et la nécessité de combiner rapidement plusieurs antihypertenseurs chez la majorité des patients hypertendus. En effet, elles mentionnent qu'une monothérapie n'est efficace que chez un nombre limité de patients, et que d'associer deux molécules serait même plus efficace que de monter la dose de la monothérapie (surtout chez les patients hypertendus sévères ou à risques cardiovasculaires élevés). En cas d'échec de la bithérapie ou de contrôle tensionnel insuffisant, l'algorithme thérapeutique recommandé préconise ensuite d'augmenter le dosage de la bithérapie initiale, ou de passer à une autre bithérapie, ou de prescrire une trithérapie. En cas d'utilisation d'une bi- ou trithérapie, les recommandations européennes favorisent la prise unique, car réduire le nombre de prises par jour ainsi que le nombre de comprimés améliore la compliance et le contrôle tensionnel.

En Belgique, il y a peu de données épidémiologiques disponibles concernant le traitement actuel de l'hypertension artérielle et comment ces recommandations récentes sont traduites dans la pratique courante des médecins-généralistes. La présente étude essaie de remédier à cette situation.

2. Objectif

L'objectif de la présente étude est d'évaluer, dans la pratique courante des médecins généralistes en Belgique, l'atteinte du contrôle tensionnel cible chez les patients hypertendus traités par plusieurs antihypertenseurs suivant le type de traitement reçu.

En particulier, l'étude s'intéresse à évaluer le taux de contrôle tensionnel suivant

- le nombre d'antihypertenseurs pris (deux ou trois),
- les différentes combinaisons d'antihypertenseurs utilisées,
- le type d'association (libre ou fixe, recommandée ou non).

Valeur ajoutée attendue

La réalisation de cette étude devrait permettre

- d'avoir une meilleure idée de l'efficacité réelle en pratique clinique courante de l'association de plusieurs antihypertenseurs comme traitement de l'hypertension artérielle,
- d'identifier des lacunes dans l'approche thérapeutique de ces patients,
- de proposer des pistes d'actions pour améliorer leur contrôle tensionnel.

3. Design

La méthodologie utilisée sera celle d'une étude observationnelle transversale (cross-sectional survey).

Ce plan d'étude a les caractéristiques suivantes :

- Seules des données existantes recueillies habituellement en pratique quotidienne sont collectées. Ces données de routine ne sont pas recueillies spécifiquement pour répondre à l'objectif de l'étude.
- Les données seront collectées à un seul instant précis dans le temps, permettant ainsi d'avoir une vue transversale sur la population étudiée. Dans ce cas précis, l'étude s'intéresse à la situation actuelle (en 2015).
- L'étude est purement descriptive (l'analyse statistique peut décrire la situation actuelle), mais ne permet pas d'étudier l'évolution de cette situation dans le temps (seulement possible par une étude longitudinale).

Il est parfaitement adapté à répondre aux questions posées.

Partant de données déjà disponibles et n'impliquant pas les patients concernés, ce type d'étude est considéré comme «rétrospective» dans le sens du paragraphe 1.4 du Guide d'évaluation des études non-interventionnelles de mai 2008 [(2)], et ne relève donc pas du champ d'application de la loi relative aux expérimentations sur la personne humaine (7 mai 2004). Dans ce cadre, l'étude a obtenu un visa préalable du Bureau des visas de pharma.be, suivant les recommandations émises dans son Code de déontologie (23 mars 2012).

4. Patients et investigateurs

4.1 Patients

Les patients visés par l'étude sont des patients avec hypertension artérielle déjà traités par plusieurs antihypertenseurs, consultant un médecin-généraliste en Belgique.

Le nombre de patients dont les données seront collectées est fixé à 5000.

Ce nombre n'est pas basé sur un calcul statistique, ce qui est inhérent au type de plan d'étude utilisé, mais est comparable à la taille d'échantillon courante pour ce type d'enquête épidémiologique.

4.2 Investigateurs

Les investigateurs visés par l'étude sont des médecins-généralistes, pratiquant en Belgique, et qui voient couramment le type de patients visés dans leurs consultations.

Le nombre de médecins nécessaire à collecter les données de 5000 patients est estimé à 420, chaque généraliste collectant ainsi les données de 12 patients éligibles vus en consultation. Ces médecins-généralistes sont recrutés dans l'ensemble de la Belgique, avec une représentation géographique équilibrée, au cours d'une période de 4 mois (du 19 janvier 2015 au 19 mai 2015), et ce afin de permettre aux informateurs médicaux de Servier Benelux de mettre sur pied les rendez-vous avec les investigateurs pour les visites initiales, d'obtenir leur accord de participation et la signature du contrat, et de remettre le matériel nécessaire pour l'initiation de l'étude.

Afin de ne pas introduire de biais de sélection de patients, il est demandé aux investigateurs d'inclure les 12 derniers patients consécutifs répondant aux critères de l'étude qui se sont présentés récemment à leur consultation.

5. Données collectées

Pour chaque patient éligible, les données existantes suivantes sont collectées par l'investigateur sur un formulaire d'observation :

- Le n° du patient dans l'étude (de 1 à 12), son âge et sexe.
- Hypertension artérielle : compliquée (diabète, antécédents cardiovasculaires, insuffisance rénale) ou non, nombre d'antihypertenseurs pris.
- Pression artérielle systolique/diastolique (mm Hg), estimée contrôlée ou non.
- Traitement médicamenteux : antihypertenseurs pris, posologie, type d'association (libre ou fixe).
- Raison du choix thérapeutique.

Mise à part les données patients, le formulaire d'observation récolte également le nom de l'investigateur, son adresse, sa signature et son cachet, afin de pouvoir le contacter en cas de besoin, et comme garantie d'authenticité.

Le formulaire d'observation mentionne le numéro de visa de pharma.be ainsi que le nom de la personne de contact chez Servier Benelux, afin de permettre la demande d'informations complémentaires en cas de besoin.

Les données nécessaires à l'étude sont collectées au cours d'une période de 3 mois suivant l'accord de l'investigateur de participer à l'étude, afin de laisser à ces généralistes à l'emploi du temps chargé le temps nécessaire pour identifier les 12 patients éligibles et pour remplir les formulaires d'observation correspondants. Ensuite, les visiteurs médicaux de Servier Benelux auront 2 mois pour recueillir les formulaires d'observation. Vu que la date limite de recrutement des investigateurs sera le 19 mai 2015, toutes les données collectées devraient être disponibles au 19 août 2015, date de fin d'étude.

Si l'investigateur, au cours de sa recherche portant sur les données à récolter pour l'étude identifierait dans le dossier d'un patient, un effet indésirable potentiellement lié à la prise d'un des médicaments de Servier Benelux qui n'a pas (encore) été notifié à Servier Benelux, il est demandé de le faire dans le plus bref délai en complétant le formulaire de Rapport d'effet indésirable et de l'envoyer immédiatement au responsable de la pharmacovigilance de Servier Benelux, le Dr Xavier Pottier (fax : 025294389 , e-mail : pharmacovigilance@be.netgrs.com, tél : 025294311).

Il est également demandé à l'investigateur de notifier à Servier Benelux toutes les situations particulières en relation avec l'un des médicaments de Servier Benelux, c'est-à-dire :

- tout événement indésirable entraînant l'arrêt du traitement,
- toute suspicion de transmission d'un agent infectieux via le médicament,
- tout surdosage (intentionnel ou accidentel), abus ou mésusage,
- toute utilisation hors-indication (off-label),
- toute erreur médicamenteuse,
- toute absence de l'effet pharmacologique attendu (manque d'efficacité) pour les produits utilisés dans les maladies potentiellement mortelles à court terme,
- toute exposition au médicament pendant la grossesse ou l'allaitement,
- toute exposition professionnelle,
- tout événement indésirable lié à un défaut de qualité du produit ou une contrefaçon.

Pour notifier les situations particulières en relation avec l'un des médicaments de Servier Benelux, le formulaire de Rapport d'effets indésirables est également à utiliser.

Si besoin, le responsable de la pharmacovigilance de Servier Benelux contactera l'investigateur afin d'obtenir plus de détails.

En cas d'effets indésirables avec un autre médicament, nous vous recommandons d'en informer l'Agence Fédérale des Médicaments et Produits de Santé via le site www.fagg-afmps.be ou le titulaire du médicament.

6. Analyse des résultats

Après un contrôle de qualité, les données de tous les formulaires d'observation correctement remplis seront entrées dans un tableau Excel permettant les analyses statistiques descriptives suivantes :

- Population étudiée : nombre de patients, répartition suivant leur âge et sexe, pourcentage de patients avec HTA compliquée, répartition suivant le nombre d'antihypertenseurs pris.
- Pression artérielle : systolique et diastolique (population globale versus sous-population à risque), taux de bon contrôle tensionnel (global vs patients à risques, suivant la sévérité de l'hypertension, suivant le nombre d'antihypertenseurs pris, suivant les différentes combinaisons d'antihypertenseurs utilisées, suivant le type d'association).
- Traitement médicamenteux : répartition suivant le nombre d'antihypertenseurs pris, les différentes combinaisons utilisées, le type d'association (libre ou fixe, recommandée ou non), la raison du choix thérapeutique.

La gestion des données, ainsi que les analyses statistiques, seront réalisées par la société de services PharmaCS (Heirbaan 26, 1785 Merchtem), sous la supervision du service scientifique de Servier Benelux.

7. Rapport d'étude et publication

Après l'analyse des données et l'interprétation des résultats, un rapport d'étude sera rédigé par PharmaCS (Heirbaan 26, 1785 Merchtem) et approuvé par le service scientifique de Servier Benelux. Une publication dans une revue scientifique sera également envisagée. Ce rapport (ou publication) sera remis(e) à chacun des investigateurs ayant participé à l'étude. Il (elle) sera également mis(e) à la disposition des organes de pharma.be énoncés à l'article 52, §1 de son Code de déontologie dd 23 mars 2012.

8. Gestion de qualité

Le protocole d'étude a été élaboré en collaboration avec le service scientifique de Servier Benelux, qui l'a ensuite approuvé, et qui veillera au bon déroulement de l'étude.

L'étude a reçu un visa préalable du Bureau des visas de pharma.be, suivant les recommandations émises dans son Code de déontologie (23 mars 2012).

Afin de garantir la qualité des données récoltées, un contrôle de qualité sera organisé, qui sera adapté aux risques de qualité inhérents à la méthodologie observationnelle de l'étude. À cette fin, les formulaires d'observation seront vérifiés par le service scientifique de Servier Benelux chez un échantillon de 5% des investigateurs ayant participé à l'étude.

La gestion des données, les analyses statistiques, ainsi que la rédaction du rapport d'étude seront réalisées par PharmaCS (Heirbaan 26, 1785 Merchtem), sous la supervision du service scientifique de Servier Benelux.

Le rôle des informateurs médicaux de Servier Benelux sera limité aux deux actions suivantes :

- Visite d'initiation : présentation de l'étude, remise du protocole et du formulaire d'observation, signature du contrat avec l'investigateur.
- Visite de clôture : reprise des formulaires d'observations complétés.

9. Références

1. Mancia G, Fagard R et al. 2013 ESH/ESC Guidelines for the management of arterial hypertension. Journal of Hypertension 2013, 31: 1281-1357.
2. Bogaert M et al. Guide d'évaluation des études non interventionnelles. http://www.faggafmps. be/fr/humain/medicaments/medicaments/recherche_developpement/comite_d_e thique/

10. Signatures

INVESTIGATEUR

Nom : . ...

Signature :

SERVIER BENELUX

Nom : Pauwels Valerie

Signature :

Patient	Date de collecte des données (jj/mm/aaaa)	Age (ans)	Sexe (H/F)	Hypertension compliquée ?	PAS/PAD (mmHg)	Patient contrôlé ?	Traitement antihypertenseur actuel :	
1				☐ Diabète<br>☐ Antécédents CV<br>☐ Insuffisance rénale<br>☐ Autres		☐ Oui<br>☐ Non	**En association libre :**<br>(Produit / Posologie)<br>................ / ....<br>................ / ....<br>................ / ....<br>**En association fixe :**<br>(Produit / Posologie)<br>................ / ....<br>................ / ....<br>................ /	Dans le cas où le patient ne prend pas d’association fixe de deux ou trois produits, envisageriez-vous de switcher son traitement vers<br>**une bithérapie fixe :** ☐ Oui ☐ Non<br>**une trithérapie fixe :** ☐ Oui ☐ Non<br>Si oui pour quelle raison ?<br>(plusieurs choix possibles)<br>☐ Meilleure compliance<br>☐ Meilleure contrôle tensionnel<br>☐ Meilleur pronostic<br>☐ Autre :
2				☐ Diabète<br>☐ Antécédents CV<br>☐ Insuffisance rénale<br>☐ Autres		☐ Oui<br>☐ Non	**En association libre :**<br>(Produit / Posologie)<br>................ / ....<br>................ / ....<br>................ / ....<br>**En association fixe :**<br>(Produit / Posologie)<br>................ / ....<br>................ / ....<br>................ /	Dans le cas où le patient ne prend pas d’association fixe de deux ou trois produits, envisageriez-vous de switcher son traitement vers<br>**une bithérapie fixe :** ☐ Oui ☐ Non<br>**une trithérapie fixe :** ☐ Oui ☐ Non<br>Si oui pour quelle raison ?<br>(plusieurs choix possibles)<br>☐ Meilleure compliance<br>☐ Meilleure contrôle tensionnel<br>☐ Meilleur pronostic<br>☐ Autre :
3				☐ Diabète<br>☐ Antécédents CV<br>☐ Insuffisance rénale<br>☐ Autres		☐ Oui<br>☐ Non	**En association libre :**<br>(Produit / Posologie)<br>................ / ....<br>................ / ....<br>................ / ....<br>**En association fixe :**<br>(Produit / Posologie)<br>................ / ....<br>................ / ....<br>................ /	Dans le cas où le patient ne prend pas d’association fixe de deux ou trois produits, envisageriez-vous de switcher son traitement vers<br>**une bithérapie fixe :** ☐ Oui ☐ Non<br>**une trithérapie fixe :** ☐ Oui ☐ Non<br>Si oui pour quelle raison ?<br>(plusieurs choix possibles)<br>☐ Meilleure compliance<br>☐ Meilleure contrôle tensionnel<br>☐ Meilleur pronostic<br>☐ Autre :
4				☐ Diabète<br>☐ Antécédents CV<br>☐ Insuffisance rénale<br>☐ Autres		☐ Oui<br>☐ Non	**En association libre :**<br>(Produit / Posologie)<br>................ / ....<br>................ / ....<br>................ / ....<br>**En association fixe :**<br>(Produit / Posologie)<br>................ / ....<br>................ / ....<br>................ /	Dans le cas où le patient ne prend pas d’association fixe de deux ou trois produits, envisageriez-vous de switcher son traitement vers<br>**une bithérapie fixe :** ☐ Oui ☐ Non<br>**une trithérapie fixe :** ☐ Oui ☐ Non<br>Si oui pour quelle raison ?<br>(plusieurs choix possibles)<br>☐ Meilleure compliance<br>☐ Meilleure contrôle tensionnel<br>☐ Meilleur pronostic<br>☐ Autre :

Patient	Date de collecte des données (jj/mm/aaaa)	Age (ans)	Sexe (H/F)	Hypertension compliquée ?	PAS/PAD (mmHg)	Patient contrôlé ?	Traitement antihypertenseur actuel :	
5				☐ Diabète<br>☐ Antécédents CV<br>☐ Insuffisance rénale<br>☐ Autres		☐ Oui<br>☐ Non	**En association libre :**<br>(Produit / Posologie)<br>................ / ....<br>................ / ....<br>................ / ....<br>**En association fixe :**<br>(Produit / Posologie)<br>................ / ....<br>................ / ....<br>................ /	Dans le cas où le patient ne prend pas d’association fixe de deux ou trois produits, envisageriez-vous de switcher son traitement vers<br>**une bithérapie fixe :** ☐ Oui ☐ Non<br>**une trithérapie fixe :** ☐ Oui ☐ Non<br>Si oui pour quelle raison ?<br>(plusieurs choix possibles)<br>☐ Meilleure compliance<br>☐ Meilleure contrôle tensionnel<br>☐ Meilleur pronostic<br>☐ Autre :
6				☐ Diabète<br>☐ Antécédents CV<br>☐ Insuffisance rénale<br>☐ Autres		☐ Oui<br>☐ Non	**En association libre :**<br>(Produit / Posologie)<br>................ / ....<br>................ / ....<br>................ / ....<br>**En association fixe :**<br>(Produit / Posologie)<br>................ / ....<br>................ / ....<br>................ /	Dans le cas où le patient ne prend pas d’association fixe de deux ou trois produits, envisageriez-vous de switcher son traitement vers<br>**une bithérapie fixe :** ☐ Oui ☐ Non<br>**une trithérapie fixe :** ☐ Oui ☐ Non<br>Si oui pour quelle raison ?<br>(plusieurs choix possibles)<br>☐ Meilleure compliance<br>☐ Meilleure contrôle tensionnel<br>☐ Meilleur pronostic<br>☐ Autre :
7				☐ Diabète<br>☐ Antécédents CV<br>☐ Insuffisance rénale<br>☐ Autres		☐ Oui<br>☐ Non	**En association libre :**<br>(Produit / Posologie)<br>................ / ....<br>................ / ....<br>................ / ....<br>**En association fixe :**<br>(Produit / Posologie)<br>................ / ....<br>................ / ....<br>................ /	Dans le cas où le patient ne prend pas d’association fixe de deux ou trois produits, envisageriez-vous de switcher son traitement vers<br>**une bithérapie fixe :** ☐ Oui ☐ Non<br>**une trithérapie fixe :** ☐ Oui ☐ Non<br>Si oui pour quelle raison ?<br>(plusieurs choix possibles)<br>☐ Meilleure compliance<br>☐ Meilleure contrôle tensionnel<br>☐ Meilleur pronostic<br>☐ Autre :
8				☐ Diabète<br>☐ Antécédents CV<br>☐ Insuffisance rénale<br>☐ Autres		☐ Oui<br>☐ Non	**En association libre :**<br>(Produit / Posologie)<br>................ / ....<br>................ / ....<br>................ / ....<br>**En association fixe :**<br>(Produit / Posologie)<br>................ / ....<br>................ / ....<br>................ /	Dans le cas où le patient ne prend pas d’association fixe de deux ou trois produits, envisageriez-vous de switcher son traitement vers<br>**une bithérapie fixe :** ☐ Oui ☐ Non<br>**une trithérapie fixe :** ☐ Oui ☐ Non<br>Si oui pour quelle raison ?<br>(plusieurs choix possibles)<br>☐ Meilleure compliance<br>☐ Meilleure contrôle tensionnel<br>☐ Meilleur pronostic<br>☐ Autre :

N’oubliez pas de remplir vos coordonnées

Patient	Date de collecte des données (jj/mm/aaaa)	Age (ans)	Sexe (H/F)	Hypertension compliquée ?	PAS/PAD (mmHg)	Patient contrôlé ?	Traitement antihypertenseur actuel :	
9				☐ Diabète<br>☐ Antécédents CV<br>☐ Insuffisance rénale<br>☐ Autres		☐ Oui<br>☐ Non	**En association libre :**<br>(Produit / Posologie)<br>................ / ....<br>................ / ....<br>................ / ....<br>**En association fixe :**<br>(Produit / Posologie)<br>................ / ....<br>................ / ....<br>................ /	Dans le cas où le patient ne prend pas d'association fixe de deux ou trois produits, envisageriez-vous de switcher son traitement vers<br>**une bithérapie fixe :** ☐ Oui ☐ Non<br>**une trithérapie fixe :** ☐ Oui ☐ Non<br>Si oui pour quelle raison ?<br>(plusieurs choix possibles)<br>☐ Meilleure compliance<br>☐ Meilleure contrôle tensionnel<br>☐ Meilleur pronostic<br>☐ Autre :
10				☐ Diabète<br>☐ Antécédents CV<br>☐ Insuffisance rénale<br>☐ Autres		☐ Oui<br>☐ Non	**En association libre :**<br>(Produit / Posologie)<br>................ / ....<br>................ / ....<br>................ / ....<br>**En association fixe :**<br>(Produit / Posologie)<br>................ / ....<br>................ / ....<br>................ /	Dans le cas où le patient ne prend pas d'association fixe de deux ou trois produits, envisageriez-vous de switcher son traitement vers<br>**une bithérapie fixe :** ☐ Oui ☐ Non<br>**une trithérapie fixe :** ☐ Oui ☐ Non<br>Si oui pour quelle raison ?<br>(plusieurs choix possibles)<br>☐ Meilleure compliance<br>☐ Meilleure contrôle tensionnel<br>☐ Meilleur pronostic<br>☐ Autre :
11				☐ Diabète<br>☐ Antécédents CV<br>☐ Insuffisance rénale<br>☐ Autres		☐ Oui<br>☐ Non	**En association libre :**<br>(Produit / Posologie)<br>................ / ....<br>................ / ....<br>................ / ....<br>**En association fixe :**<br>(Produit / Posologie)<br>................ / ....<br>................ / ....<br>................ /	Dans le cas où le patient ne prend pas d'association fixe de deux ou trois produits, envisageriez-vous de switcher son traitement vers<br>**une bithérapie fixe :** ☐ Oui ☐ Non<br>**une trithérapie fixe :** ☐ Oui ☐ Non<br>Si oui pour quelle raison ?<br>(plusieurs choix possibles)<br>☐ Meilleure compliance<br>☐ Meilleure contrôle tensionnel<br>☐ Meilleur pronostic<br>☐ Autre :
12				☐ Diabète<br>☐ Antécédents CV<br>☐ Insuffisance rénale<br>☐ Autres		☐ Oui<br>☐ Non	**En association libre :**<br>(Produit / Posologie)<br>................ / ....<br>................ / ....<br>................ / ....<br>**En association fixe :**<br>(Produit / Posologie)<br>................ / ....<br>................ / ....<br>................ /	Dans le cas où le patient ne prend pas d'association fixe de deux ou trois produits, envisageriez-vous de switcher son traitement vers<br>**une bithérapie fixe :** ☐ Oui ☐ Non<br>**une trithérapie fixe :** ☐ Oui ☐ Non<br>Si oui pour quelle raison ?<br>(plusieurs choix possibles)<br>☐ Meilleure compliance<br>☐ Meilleure contrôle tensionnel<br>☐ Meilleur pronostic<br>☐ Autre :

En cas d'effets indésirables ou toutes situations particulières en relation avec un médicament, veuillez vous référer au paragraphe 5 du protocole.

ACHIEVE

Cross-sectional survey evaluating blood pressure target **ACHIEVE**ment in the Belgian general hypertensive population treated with multiple anti-hypertensive agents according to the treatment received

ACHIEVE IC4-06593-012-BEL
Formulaire d'observation approuvé le 25/11/14 - BF 15 TM C2 BI LP 01
Visa Pharma.be : VI 14/10/06/01 obtenu le 16/10/2014

Etude observationnelle transversale du taux de contrôle tensionnel dans la population générale de patients hypertendus en Belgique traités par plusieurs antihypertenseurs suivant le type de traitement reçu

Personne de contact Servier : ....

Nom de l'investigateur : ....

Adresse : ....
....

Signature :

Cachet
