# Supplementary material for: Cross-sectional survey evaluating blood pressure control ACHIEVEment in hypertensive patients treated with multiple anti-hypertensive agents in Belgium and Luxembourg
Source: PLoS One. 2018 Nov 1;13(11):e0206510. doi: 10.1371/journal.pone.0206510 (PMC6211697; doi:10.1371/journal.pone.0206510)
Supplement: S2 File — Investigateur. Amendment to the ACHIEVE protocol justifying the 2nd inclusion wave of patients. (PDF) [file pone.0206510.s002.pdf]

# ACHIEVE

## Cross-sectional survey evaluating blood pressure target ACHIEVEment in the Belgian general hypertensive population treated with multiple anti-hypertensive agents according to the treatment received

ACHIEVE IC4-06593-012-BEL Visa Pharma.be : VI 14/10/06/01 obtenu le 16/10/2014



**Etude observationnelle transversale du taux de contrôle tensionnel dans la population générale de patients hypertendus en Belgique traités par plusieurs antihypertenseurs suivant le type de traitement reçu**

# Amendement 1 au Protocole d'étude

## 1. Justification de l'amendement

Dans le protocole initial, il était prévu de recruter 420 médecins généralistes dans l'étude, qui collecteraient chacun certaines données existantes de 12 patients éligibles, afin de pouvoir analyser les données recherchées de 5000 patients.
Ces données devaient être disponibles au 19 août 2015.

A la date du 25 août 2015, 327 formulaires d'observation ont été récupérés, et les données de 3288 patients ont été rentrées dans la banque de données de l'étude.
Une analyse préliminaire des données disponibles fait apparaître des résultats intéressants. Ils ont été présentés et discutés avec un médecin spécialiste en hypertension, qui trouve qu'ils mériteraient d'être explorés à plus large échelle.

En effet, les auteurs d'une étude observationnelle transversale récente similaire sur la prévalence de l'hypertension systolique chez des patients hypertendus traités par des médecins généralistes en Belgique (étude I-inSYST, réalisée par P. Van der Niepen, C. Giot et Ph. van de Borne, réf. 1), ont contacté plus de 1000 médecins généralistes afin de pouvoir collecter les données de 10000 patients. Les résultats de cette étude ont été publiés dans le Journal of Hypertension.
Elargir l'étude de 5000 à 10000 patients,
- augmenterait la précision de l'estimation des pourcentages et proportions des variables étudiées, avec une marge d'erreur de moins de 1 % (réf. 2), ce qui augmenterait la robustesse des conclusions,
- et faciliterait la présentation des résultats à un congrès européen, ainsi que leur publication dans une revue médicale internationale.

Dès lors, il est proposé de :

- prolonger la période de recueil des données jusqu'au 19 septembre 2015, afin de rattraper au mieux le retard et d'arriver le plus proche possible des 5000 patients prévus;
- réaliser une deuxième phase d'étude et de recruter 420 médecins généralistes supplémentaires durant une période de 4 mois (du 14 octobre 2015 au 14 février 2016), et de donner à chaque médecin généraliste une période de 3 mois pour collecter les données demandées de 12 patients éligibles. Ensuite, les visiteurs médicaux de Servier Benelux auront 3 mois pour recueillir les formulaires d'observation.

La date limite pour rentrer les données dans la banque de données, et date de fin d'étude serait alors le 14 septembre 2016.

# 2. Texte de l'amendement

Les phrases ou paragraphes suivants surlignés en jaune sont à ajouter aux Sections 4 et 5 du protocole d'étude initial :

## 4. Patients et investigateurs

### 4.1 Patients

[…]

Le nombre de patients dont les données seront collectées est fixé à 5000. Dans une deuxième phase de l'étude les données de 5000 patients supplémentaires seront collectées.

[…]

### 4.2 Investigateurs

Les investigateurs visés par l'étude sont des médecins-généralistes, pratiquant en Belgique, et qui voient couramment le type de patients visés dans leurs consultations.

Le nombre de médecins nécessaire à collecter les données de 5000 patients est estimé à 420, chaque généraliste collectant ainsi les données de 12 patients éligibles vus en consultation. Ces médecins-généralistes sont recrutés dans l'ensemble de la Belgique, avec une représentation géographique équilibrée, au cours d'une période de 4 mois (du 19 janvier 2015 au 19 mai 2015), et ce afin de permettre aux informateurs médicaux de Servier Benelux de mettre sur pied les rendez-vous avec les investigateurs pour les visites initiales, d'obtenir leur accord de participation et la signature du contrat, et de remettre le matériel nécessaire pour l'initiation de l'étude.

Pour la deuxième phase de l'étude, 420 médecins généralistes supplémentaires seront recrutés en Belgique, qui collecteront chacun les données de 12 patients éligibles vus en consultation. Le recrutement des investigateurs pour la deuxième phase aura lieu durant une période de 4 mois (du 14 octobre 2015 au 14 février 2016).

Afin de ne pas introduire de biais de sélection de patients, il est demandé aux investigateurs d'inclure les 12 derniers patients consécutifs répondant aux critères de l'étude qui se sont présentés récemment à leur consultation.

## 5. Données collectées

Pour chaque patient éligible, […]

Les données nécessaires à l'étude sont collectées au cours d'une période de 3 mois suivant l'accord de l'investigateur de participer à l'étude, afin de laisser à ces généralistes à l'emploi du temps chargé le temps nécessaire pour identifier les 12 patients éligibles et pour remplir les formulaires d'observation correspondants. Ensuite, les visiteurs médicaux de Servier Benelux auront 2 mois pour recueillir les formulaires d'observation. Vu que la date limite de recrutement des investigateurs sera le 19 mai 2015, toutes les données collectées devraient être disponibles au 19 août 2015, date de fin d'étude.

Vu le retard dans la collecte des données de la première phase de l'étude, cette collecte ainsi que la rentrée des données dans la banque de données de l'étude sera prolongée jusqu'au 19 septembre 2015.

Pour la deuxième phase de l'étude, le timing suivant est proposé :

- date limite de recrutement des investigateurs : le 14 février 2016,
- date limite pour les investigateurs de remplir les formulaires d'observation : le 14 mai 2016,
- date limite pour récupérer les formulaires d'observation : le 14 août 2016,
- date limite pour rentrer les données dans la banque de données, et date de fin d'étude : le 14 septembre 2016.

Si l'investigateur, […]

# 3. Références

1. Van der Niepen P., Giot C. & van de Borne Ph. Prevalence of isolated uncontrolled systolic blood pressure among treated hypertensive patients in primary care in Belgium: results of the I-inSYST survey. *Journal of Hypertension, 26, 2057-2063, 2008.*

2. Feiss J.L. Statistical methods for rates and proportions, 2nd edition, Wiley, New York, 13-15, 1981.

# 4. Signatures

INVESTIGATEUR

Nom : ....................................

Signature :

SERVIER BENELUX

Nom : Pauwels Valerie

Signature :
