# Supplementary material for: Cross-sectional survey evaluating blood pressure control ACHIEVEment in hypertensive patients treated with multiple anti-hypertensive agents in Belgium and Luxembourg
Source: PLoS One. 2018 Nov 1;13(11):e0206510. doi: 10.1371/journal.pone.0206510 (PMC6211697; doi:10.1371/journal.pone.0206510)
Supplement: S3 File — Scientific protocol and CRF of the ACIEVE study in english. (PDF) [file pone.0206510.s003.pdf]

ACHIEVE

Evaluation of achieved blood pressure control with more than one antihypertensive drug in patients treated by general practitioners in Belgium

Study protocol

1. scientific rationale

High blood pressure is still a major cause of death and one of the main influencing risk factors for heart, cerebrovascular and renovascular diseases. Despite the impact of this disease on public health and healthcare costs, its diagnosis is often ignored and its treatment remains sub-optimal. It is estimated that at least half of hypertensive patients are not known, at least half of patients treated remain uncontrolled, and half of patients treated with antihypertensive drugs do not adhere to long-term treatment.
General practitioners play a vital role in its early diagnosis and proper treatment.

In addition to non-pharmacological measures for the prevention and treatment of hypertension, the recent recommendations of the European Society of Hypertension and the European Society of Cardiology of 2013(1) recommend that pharmacological treatment be started rapidly.

Pharmacological management uses the currently available classes of antihypertensive agents, i.e. thiazide diuretics, beta-blockers, calcium antagonists, angiotensin conversion enzyme inhibitors and angiotensin receptor antagonists. In addition, these recommendations highlight the importance and necessity of rapidly combining several antihypertensive agents in the majority of hypertensive patients. Indeed, they mention that monotherapy is only effective in a limited number of patients, and that combining two molecules would be even more effective than increasing the dose of monotherapy (especially in patients with severe hypertension or high cardiovascular risk). In case of failure of the dual therapy or insufficient blood pressure control, the recommended therapeutic algorithm then recommends increasing the dosage of the initial dual therapy, or switching to another dual therapy, or prescribing triple therapy. When using bi- or tritherapy, the European recommendations favour a single dose, because reducing the number of doses per day as well as the number of tablets improves compliance and blood pressure control.

In Belgium, there is little epidemiological data available on the current treatment of high blood pressure and how these recent recommendations are translated into the current practice of general practitioners. This study attempts to remedy this situation.

2. Objectif

The objective of this study is to evaluate, in the current practice of general practitioners in Belgium, the achievement of target blood pressure control in hypertensive patients treated with several antihypertensive drugs depending on the type of treatment received.
In particular, the study is interested in evaluating the following rate of blood pressure control

- the number of antihypertensive molcules takne (2 or 3),
- the different combinations of antihypertensive drugs taken,
- the type of association (free, fixed or both free and fixed)

Expected added value

The realisation of this study should allow

- to have a better understanding of the actual effectiveness in current clinical practice of combining multiple antihypertensive agents as a treatment for high blood pressure,
- to identify gaps in the therapeutic approach of these patients,
- to propose courses of action to improve blood pressure control.

3. Design

The methodology used will be a cross-sectional survey. This study design has the following characteristics:

- Only existing data usually collected in daily practice are collected. These routine data are not collected specifically to meet the study objective.
- data will be collected at a single precise moment in time, thus providing a cross-sectional view of the population studied. In this specific case, the study looks at the current situation (in 2015).
- The study is purely descriptive (the statistical analysis can describe the current situation), but does not allow to study the evolution of this situation over time (only possible by a longitudinal study).

it is perfectly suited to answering the questions asked.

On the basis of data already available and not involving the patients concerned, this type of study is considered'retrospective' within the meaning of paragraph 1.4 of the Guide to the Evaluation of Non-Interventional Studies of May 2008 (2), and therefore does not fall within the scope of the Human Experiments Act (7 May 2004). In this context, the study obtained a prior visa from the Visa Office of pharma.be, following the recommendations made in its Code of Ethics (23 March 2012).

4. Patients et investigators

4.1 Patients

The patients targeted by the study are patients with high blood pressure already treated with several antihypertensives, consulting a general practitioner in Belgium. .

The number of patients whose data will be collected is set at 5000.

This number is not based on a statistical calculation, which is inherent in the type of study design used, but is comparable to the current sample size for this type of epidemiological survey.

4.2 Investigators

The investigators targeted by the study are general practitioners, practicing in Belgium, who commonly see the type of patients targeted in their consultations.

The number of doctors needed to collect data from 5000 patients is estimated at 420, each GP collecting data from 12 eligible patients seen in consultation. These general practitioners are recruited throughout Belgium, with a balanced geographical representation, over a period of 4 months (from 19 January 2015 to 19 May 2015), in order to enable the medical informants of Servier Benelux to set up appointments with the investigators for the initial visits, to obtain their agreement to participate and to sign the contract, and to submit the necessary material for the initiation of the study.

In order not to introduce patient selection bias, investigators are asked to include the last 12 consecutive patients meeting the study criteria who recently presented for consultation.

5. Collected data

For each eligible patient, the following existing data are collected by the investigator on an observation form:

- The number of the patient in the study (from 1 to 12), his age and sex.
- Hypertension: complicated (diabetes, cardiovascular history, renal failure) or not, number of antihypertensive drugs taken.
- systolic/diastolic (mm Hg) blood pressure, estimated controlled or uncontrolled.
- Drug treatment: antihypertensives taken, dosage, type of combination (free or fixed).
- Reason for therapeutic choice

Apart from the patient data, the observation form also collects the investigator's name, address, signature and stamp, so that it can be contacted if necessary, and as a guarantee of authenticity.

The observation form mentions the visa number of pharma.be and the name of the contact person at Servier Benelux, so that additional information can be requested if necessary.

The data required for the study are collected over a 3-month period following the investigator's agreement to participate in the study, in order to allow these busy general practitioners time to identify the 12 eligible patients and to complete the corresponding observation forms. Then, the medical sales representatives of Servier Benelux will have 2 months to collect the observation forms. As the deadline for recruitment of investigators will be 19 May 2015, all data collected should be available by 19 August 2015, the end date of the study.

If the investigator, in the course of researching the data to be collected for the study, identifies in a patient's file an adverse reaction potentially related to the use of one of Servier Benelux's medicines that has not (yet) been reported to Servier Benelux, it is requested to do so as soon as possible by completing the Adverse Reaction Report form and sending it immediately to Servier Benelux's pharmacovigilance Manager, Dr Xavier Pottier (fax : 025294389, e-mail : pharmacovigilance@be.netgrs.com, tel: 025294311).

The investigator is also requested to notify Servier Benelux of all special situations relating to one of Servier Benelux's medicinal products, i.e. :

- any adverse events leading to discontinuation of treatment,,
- any suspicion of transmission of an infectious agent via the drug,
- any overdose (intentional or accidental), abuse or misuse,
- any off-label use,
- any medication errors,
- any absence of the expected pharmacological effect (lack of efficacy) for products used in life-threatening diseases in the short term,
- any exposure to the drug during pregnancy or lactation,
- any occupational exposure,
- any adverse event related to a product quality defect or counterfeiting.

The Adverse Reaction Report form should also be used to report special situations in relation to one of Servier Benelux's medicines.

If necessary, the pharmacovigilance manager of Servier Benelux will contact the investigator to obtain more details.

In case of adverse reactions with another drug, we recommend that you inform the Federal Agency for Medicines and Health Products via the website www.fagg-afmps.be or the holder of the drug.

6. Analysis of the results

After quality control, data from all correctly completed observation forms will be entered into an Excel table allowing the following descriptive statistical analyses:

- Population studied: number of patients, distribution according to age and sex, percentage of patients with complicated hypertension, distribution according to the number of antihypertensive drugs taken.
- Blood pressure: systolic and diastolic (overall population versus subpopulation at risk), rate of good blood pressure control (overall vs. patients at risk, according to the severity of hypertension, according to the number of antihypertensive drugs taken, according to the different combinations of antihypertensive drugs used, according to the type of combination).
- Drug treatment: distribution according to the number of antihypertensive drugs taken, the different combinations used, the type of combination (free or fixed, recommended or not), the reason for the therapeutic choice.

Data management and statistical analysis will be carried out by the service company PharmaCS (Heirbaan 26, 1785 Merchtem), under the supervision of the scientific department of Servier Benelux.

7. Study report and publicaiton

After the data analysis and interpretation of the results, a study report will be written by PharmaCS (Heirbaan 26, 1785 Merchtem) and approved by the scientific department of Servier Benelux. Publication in a scientific journal will also be considered. This report (or publication) will be given to each investigator who participated in the study. He (she) will also be made available to the pharma.be bodies listed in article 52, §1 of its Code of Ethics dd March 23, 2012.

8. Quality control

The study protocol was drawn up in collaboration with the scientific department of Servier Benelux, which then approved it, and which will ensure that the study runs smoothly.

The study has received a prior visa from the Visa Office of pharma.be, following the recommendations made in its Code of Ethics (23 March 2012).

In order to guarantee the quality of the data collected, a quality control will be organised, which will be adapted to the quality risks inherent in the observational methodology of the study. To this end, the observation forms will be verified by the Servier Benelux scientific service in a sample of 5% of the investigators who participated in the study.

Data management, statistical analyses, as well as the drafting of the study report will be carried out by PharmaCS (Heirbaan 26, 1785 Merchtem), under the supervision of the scientific service of Servier Benelux.

The role of Servier Benelux medical informants will be limited to the following two actions:

- initiation site: presentation of the study, presentation of the protocol and the observation form, signature of the contract with the investigator.
- Closing site: return of the completed observation forms.

9. References

1. Mancia G, Fagard R et al. 2013 ESH/ESC Guidelines for the management of arterial hypertension. Journal of Hypertension 2013, 31: 1281-1357.
2. Bogaert M et al. Guide d'évaluation des études non interventionnelles. http://www.faggafmps. be/fr/humain/medicaments/medicaments/recherche_ developpement/comite_d_e thique/

10. Signatures

INVESTIGATOR

Nom : . . . .

Signature :

SERVIER BENELUX

Nom : Pauwels Valerie

Signature :

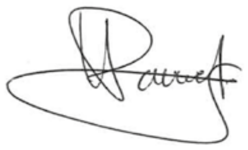

Patient	Date de collecte des données (jj/mm/aaaa)	Age (ans)	Sexe (H/F)	Hypertension compliquée ?	PAS/PAD (mmHg)	Patient contrôlé ?	Traitement antihypertenseur actuel :	
1				☐ Diabète<br>☐ Antécédents CV<br>☐ Insuffisance rénale<br>☐ Autres		☐ Oui<br>☐ Non	**En association libre :**<br>(Produit / Posologie)<br>. . . . . / . . . .<br>. . . . . / . . . .<br>. . . . . / . . . .<br>**En association fixe :**<br>(Produit / Posologie)<br>. . . . . / . . . .<br>. . . . . / . . . .<br>. . . . . /	Dans le cas où le patient ne prend pas d'association fixe de deux ou trois produits, envisageriez-vous de switcher son traitement vers<br>**une bithérapie fixe :** ☐ Oui ☐ Non<br>**une trithérapie fixe :** ☐ Oui ☐ Non<br>Si oui pour quelle raison ?<br>(plusieurs choix possibles)<br>☐ Meilleure compliance<br>☐ Meilleure contrôle tensionnel<br>☐ Meilleur pronostic<br>☐ Autre :
2				☐ Diabète<br>☐ Antécédents CV<br>☐ Insuffisance rénale<br>☐ Autres		☐ Oui<br>☐ Non	**En association libre :**<br>(Produit / Posologie)<br>. . . . . / . . . .<br>. . . . . / . . . .<br>. . . . . / . . . .<br>**En association fixe :**<br>(Produit / Posologie)<br>. . . . . / . . . .<br>. . . . . / . . . .<br>. . . . . /	Dans le cas où le patient ne prend pas d'association fixe de deux ou trois produits, envisageriez-vous de switcher son traitement vers<br>**une bithérapie fixe :** ☐ Oui ☐ Non<br>**une trithérapie fixe :** ☐ Oui ☐ Non<br>Si oui pour quelle raison ?<br>(plusieurs choix possibles)<br>☐ Meilleure compliance<br>☐ Meilleure contrôle tensionnel<br>☐ Meilleur pronostic<br>☐ Autre :
3				☐ Diabète<br>☐ Antécédents CV<br>☐ Insuffisance rénale<br>☐ Autres		☐ Oui<br>☐ Non	**En association libre :**<br>(Produit / Posologie)<br>. . . . . / . . . .<br>. . . . . / . . . .<br>. . . . . / . . . .<br>**En association fixe :**<br>(Produit / Posologie)<br>. . . . . / . . . .<br>. . . . . / . . . .<br>. . . . . /	Dans le cas où le patient ne prend pas d'association fixe de deux ou trois produits, envisageriez-vous de switcher son traitement vers<br>**une bithérapie fixe :** ☐ Oui ☐ Non<br>**une trithérapie fixe :** ☐ Oui ☐ Non<br>Si oui pour quelle raison ?<br>(plusieurs choix possibles)<br>☐ Meilleure compliance<br>☐ Meilleure contrôle tensionnel<br>☐ Meilleur pronostic<br>☐ Autre :
4				☐ Diabète<br>☐ Antécédents CV<br>☐ Insuffisance rénale<br>☐ Autres		☐ Oui<br>☐ Non	**En association libre :**<br>(Produit / Posologie)<br>. . . . . / . . . .<br>. . . . . / . . . .<br>. . . . . / . . . .<br>**En association fixe :**<br>(Produit / Posologie)<br>. . . . . / . . . .<br>. . . . . / . . . .<br>. . . . . /	Dans le cas où le patient ne prend pas d'association fixe de deux ou trois produits, envisageriez-vous de switcher son traitement vers<br>**une bithérapie fixe :** ☐ Oui ☐ Non<br>**une trithérapie fixe :** ☐ Oui ☐ Non<br>Si oui pour quelle raison ?<br>(plusieurs choix possibles)<br>☐ Meilleure compliance<br>☐ Meilleure contrôle tensionnel<br>☐ Meilleur pronostic<br>☐ Autre :

Patient	Date de collecte des données (jj/mm/aaaa)	Age (ans)	Sexe (H/F)	Hypertension compliquée ?	PAS/PAD (mmHg)	Patient contrôlé ?	Traitement antihypertenseur actuel :	
5				☐ Diabète<br>☐ Antécédents CV<br>☐ Insuffisance rénale<br>☐ Autres		☐ Oui<br>☐ Non	**En association libre :**<br>(Produit / Posologie)<br>. . . . . / . . . .<br>. . . . . / . . . .<br>. . . . . / . . . .<br>**En association fixe :**<br>(Produit / Posologie)<br>. . . . . / . . . .<br>. . . . . / . . . .<br>. . . . . /	Dans le cas où le patient ne prend pas d'association fixe de deux ou trois produits, envisageriez-vous de switcher son traitement vers<br>**une bithérapie fixe :** ☐ Oui ☐ Non<br>**une trithérapie fixe :** ☐ Oui ☐ Non<br>Si oui pour quelle raison ?<br>(plusieurs choix possibles)<br>☐ Meilleure compliance<br>☐ Meilleure contrôle tensionnel<br>☐ Meilleur pronostic<br>☐ Autre :
6				☐ Diabète<br>☐ Antécédents CV<br>☐ Insuffisance rénale<br>☐ Autres		☐ Oui<br>☐ Non	**En association libre :**<br>(Produit / Posologie)<br>. . . . . / . . . .<br>. . . . . / . . . .<br>. . . . . / . . . .<br>**En association fixe :**<br>(Produit / Posologie)<br>. . . . . / . . . .<br>. . . . . / . . . .<br>. . . . . /	Dans le cas où le patient ne prend pas d'association fixe de deux ou trois produits, envisageriez-vous de switcher son traitement vers<br>**une bithérapie fixe :** ☐ Oui ☐ Non<br>**une trithérapie fixe :** ☐ Oui ☐ Non<br>Si oui pour quelle raison ?<br>(plusieurs choix possibles)<br>☐ Meilleure compliance<br>☐ Meilleure contrôle tensionnel<br>☐ Meilleur pronostic<br>☐ Autre :
7				☐ Diabète<br>☐ Antécédents CV<br>☐ Insuffisance rénale<br>☐ Autres		☐ Oui<br>☐ Non	**En association libre :**<br>(Produit / Posologie)<br>. . . . . / . . . .<br>. . . . . / . . . .<br>. . . . . / . . . .<br>**En association fixe :**<br>(Produit / Posologie)<br>. . . . . / . . . .<br>. . . . . / . . . .<br>. . . . . /	Dans le cas où le patient ne prend pas d'association fixe de deux ou trois produits, envisageriez-vous de switcher son traitement vers<br>**une bithérapie fixe :** ☐ Oui ☐ Non<br>**une trithérapie fixe :** ☐ Oui ☐ Non<br>Si oui pour quelle raison ?<br>(plusieurs choix possibles)<br>☐ Meilleure compliance<br>☐ Meilleure contrôle tensionnel<br>☐ Meilleur pronostic<br>☐ Autre :
8				☐ Diabète<br>☐ Antécédents CV<br>☐ Insuffisance rénale<br>☐ Autres		☐ Oui<br>☐ Non	**En association libre :**<br>(Produit / Posologie)<br>. . . . . / . . . .<br>. . . . . / . . . .<br>. . . . . / . . . .<br>**En association fixe :**<br>(Produit / Posologie)<br>. . . . . / . . . .<br>. . . . . / . . . .<br>. . . . . /	Dans le cas où le patient ne prend pas d'association fixe de deux ou trois produits, envisageriez-vous de switcher son traitement vers<br>**une bithérapie fixe :** ☐ Oui ☐ Non<br>**une trithérapie fixe :** ☐ Oui ☐ Non<br>Si oui pour quelle raison ?<br>(plusieurs choix possibles)<br>☐ Meilleure compliance<br>☐ Meilleure contrôle tensionnel<br>☐ Meilleur pronostic<br>☐ Autre :

◀ *N'oubliez pas de remplir vos coordonnées*

Patient	Date de collecte des données (jj/mm/aaaa)	Age (ans)	Sexe (H/F)	Hypertension compliquée ?	PAS/PAD (mmHg)	Patient contrôlé ?	Traitement antihypertenseur actuel :	
9				☐ Diabète<br>☐ Antécédents CV<br>☐ Insuffisance rénale<br>☐ Autres		☐ Oui<br>☐ Non	**En association libre :**<br>(Produit / Posologie)<br>................ / ....<br>................ / ....<br>................ / ....<br>**En association fixe :**<br>(Produit / Posologie)<br>................ / ....<br>................ / ....<br>................ /	Dans le cas où le patient ne prend pas d'association fixe de deux ou trois produits, envisageriez-vous de switcher son traitement vers<br>**une bithérapie fixe :** ☐ Oui ☐ Non<br>**une trithérapie fixe :** ☐ Oui ☐ Non<br>Si oui pour quelle raison ?<br>(plusieurs choix possibles)<br>☐ Meilleure compliance<br>☐ Meilleure contrôle tensionnel<br>☐ Meilleur pronostic<br>☐ Autre :
10				☐ Diabète<br>☐ Antécédents CV<br>☐ Insuffisance rénale<br>☐ Autres		☐ Oui<br>☐ Non	**En association libre :**<br>(Produit / Posologie)<br>................ / ....<br>................ / ....<br>................ / ....<br>**En association fixe :**<br>(Produit / Posologie)<br>................ / ....<br>................ / ....<br>................ /	Dans le cas où le patient ne prend pas d'association fixe de deux ou trois produits, envisageriez-vous de switcher son traitement vers<br>**une bithérapie fixe :** ☐ Oui ☐ Non<br>**une trithérapie fixe :** ☐ Oui ☐ Non<br>Si oui pour quelle raison ?<br>(plusieurs choix possibles)<br>☐ Meilleure compliance<br>☐ Meilleure contrôle tensionnel<br>☐ Meilleur pronostic<br>☐ Autre :
11				☐ Diabète<br>☐ Antécédents CV<br>☐ Insuffisance rénale<br>☐ Autres		☐ Oui<br>☐ Non	**En association libre :**<br>(Produit / Posologie)<br>................ / ....<br>................ / ....<br>................ / ....<br>**En association fixe :**<br>(Produit / Posologie)<br>................ / ....<br>................ / ....<br>................ /	Dans le cas où le patient ne prend pas d'association fixe de deux ou trois produits, envisageriez-vous de switcher son traitement vers<br>**une bithérapie fixe :** ☐ Oui ☐ Non<br>**une trithérapie fixe :** ☐ Oui ☐ Non<br>Si oui pour quelle raison ?<br>(plusieurs choix possibles)<br>☐ Meilleure compliance<br>☐ Meilleure contrôle tensionnel<br>☐ Meilleur pronostic<br>☐ Autre :
12				☐ Diabète<br>☐ Antécédents CV<br>☐ Insuffisance rénale<br>☐ Autres		☐ Oui<br>☐ Non	**En association libre :**<br>(Produit / Posologie)<br>................ / ....<br>................ / ....<br>................ / ....<br>**En association fixe :**<br>(Produit / Posologie)<br>................ / ....<br>................ / ....<br>................ /	Dans le cas où le patient ne prend pas d'association fixe de deux ou trois produits, envisageriez-vous de switcher son traitement vers<br>**une bithérapie fixe :** ☐ Oui ☐ Non<br>**une trithérapie fixe :** ☐ Oui ☐ Non<br>Si oui pour quelle raison ?<br>(plusieurs choix possibles)<br>☐ Meilleure compliance<br>☐ Meilleure contrôle tensionnel<br>☐ Meilleur pronostic<br>☐ Autre :

En cas d'effets indésirables ou toutes situations particulières en relation avec un médicament, veuillez vous référer au paragraphe 5 du protocole.

ACHIEVE

Cross-sectional survey evaluating blood pressure target **ACHIEVE**ment in the Belgian general hypertensive population treated with multiple anti-hypertensive agents according to the treatment received

ACHIEVE IC4-06593-012-BEL
Formulaire d'observation approuvé le 25/11/14 - BF 15 TM C2 BI LP 01
Visa Pharma.be : VI 14/10/06/01 obtenu le 16/10/2014

Etude observationnelle transversale du taux de contrôle tensionnel dans la population générale de patients hypertendus en Belgique traités par plusieurs antihypertenseurs suivant le type de traitement reçu

Personne de contact Servier : ....

Nom de l'investigateur : ....

Adresse : ....
....

Signature :

Cachet
